# How does acute caffeine ingestion affect maximal strength and muscular power in bench press and back squat in resistance-trained men?

**DOI:** 10.1080/15502783.2025.2587791

**Published:** 2025-11-26

**Authors:** Yixuan Ma, Yihao Chen, Li Ding, Yuchun Xiao, Tze-Huan Lei, Matthew Barnes, Li Guo, Yinhang Cao, Olivier Girard

**Affiliations:** a School of Athletic Performance, Shanghai University of Sport, Shanghai, People's Republic of China; b Department of Rehabilitation Medicine, Huashan Hospital, Fudan University, Shanghai, People's Republic of China; c Physical Education Teaching and Research Department, Hunan Institute of Technology, Hengyang, People's Republic of China; d College of Physical Education, Hubei Normal University, Huangshi, People's Republic of China; e School of Sport, Exercise and Nutrition, Massey University, Palmerston North, New Zealand; f School of Exercise and Health, Shanghai University of Sport, Shanghai, People's Republic of China; g School of Human Sciences (Exercise and Sport Science), The University of Western Australia, Perth, Australia

**Keywords:** Ergogenic aid, nutritional supplement, neuromuscular function, one-maximum repetition, resistance exercise

## Abstract

**Objective:**

This study examined whether caffeine (4 mg/kg) enhances upper- and lower-body maximal strength and muscular power by increasing muscle recruitment and reducing in ratings of perceived exertion (RPE) and pain perception.

**Methods:**

Fourteen resistance-trained males completed two randomized trials involving either caffeine ingestion (4 mg/kg) or a placebo. Sixty minutes after ingesting capsule, participants performed maximal strength tests (one-maximum repetition [1RM]) followed by muscular power assessments (bar velocity and power output) at 25%, 50%, 75%, and 90% of 1RM, performing 3, 2, 1, and 1 repetitions, respectively during bench press and back squat. Outcome measured included 1RM, mean and peak velocity, mean and peak power output, surface electromyographic activity of the prime movers, RPE, and pain perception.

**Results:**

Compared to placebo, caffeine significantly increased 1RM in both bench press and back squat, while reducing RPE and pain perception and enhancing root mean square (RMS) activity in the *gluteus maximus* (all *p* < 0.05), though it did not affect the median frequency (MDF) or mean frequency (MF) in any studied muscle (all *p* > 0.05). The percent improvement in 1RM was larger (+7.0 ± 2.9% vs. + 4.1 ± 2.9%; *p* < 0.05) for the back squat than for the bench press. Furthermore, caffeine significantly increased muscular power during both exercises (all *p* < 0.05), without affecting surface electromyographic activity of the prime movers, RPE, and pain perception (all *p* > 0.05).

**Conclusion:**

Ingesting a caffeine capsule at a dose of 4 mg/kg enhances maximal strength by increasing muscle recruitment and reducing RPE and pain perception, with greater improvement observed in the bench press than in the back squat. Caffeine also improves muscular power in both exercises without altering muscle recruitment or subjective perception.

## 1 Introduction

Since the International Olympic Committee recognized caffeine as a legitimate sports supplement in 2004, its use has become widespread across various sporting disciplines [1,2]. Evidence from endurance disciplines suggests that caffeine can enhance performance by augmenting central drive, increasing motor unit firing rates, and reducing fatigue [3]. Recent studies also suggest caffeine improves resistance exercise performance, including maximal strength (one-repetition maximum [1RM]) and muscular power (bar velocity and power output) [4], though the underlying mechanisms remain unclear.

As an adenosine receptor antagonist, caffeine can reduce ratings of perceived exertion (RPE) and pain perception, which could enhance resistance exercise performance by reducing subjective discomfort [5]. Previous research revealed that caffeine (6 mg/kg) increased back squat 1RM by lowering RPE [6]. However, another study reported that while caffeine improved muscular power (bar velocity and power output) during back squats at 25−90% 1RM, it did not reduce RPE or pain perception [7]. This discrepancy may be related to the different loads used in resistance exercise. Specifically, caffeine-induced reductions in RPE and pain perception might explain gains in maximal load (1RM) but not improvements in muscular power at submaximal loads (25%-90% 1RM). Consequently, further research is needed to simultaneously evaluate the effects of caffeine on maximal strength and muscular power, and accompanying perceptual response within a single study.

Several studies have compared the effects of caffeine on upper and lower limb muscle strength [6,8,9],reporting conflicting results. Some found that caffeine (2−6 mg/kg) improved back squat 1RM but not bench press 1RM [6,10],attributing this to lower voluntary activation rates in lower limb muscles and thus greater potential for improvement [11]; however, these claim lack supporting surface electromyography (sEMG) data. Conversely, other studies found similar caffeine-induced enhancements in muscular power (25−90% 1RM) for both exercises [8,12]. These discrepancies may stem from differences in caffeine's impact on muscular qualities (maximal strength vs. muscular power) and their associated neuromuscular mechanisms. Therefore, it is necessary to compare the effects of caffeine on maximal strength and muscular power across upper and lower limbs, using sEMG to shed more light on muscle recruitment patterns.

This study aims to examine whether caffeine enhances maximal strength and muscular power by reducing RPE and pain perception, and to compare its effects on maximal strength, muscular power, and muscle recruitment between the bench press and back squat. We hypothesized that caffeine would improve both maximal strength and muscular power, but would reduce RPE and pain perception only during 1RM testing, not at submaximal loads. Additionally, we predicted that caffeine would lead to greater increases in 1RM and muscle recruitment for the back squat compared to the bench press, with no difference in muscular power between the two exercises.

## 2 Methods

### 2.1 Participants

Fourteen resistance-trained Chinese males participated in this study (age: 21 ± 2 years; height: 174 ± 5 cm; body mass: 73 ± 6 kg; resistance training experience: 4 ± 2 years). None reported using nutritional supplements in the three months prior. Habitual caffeine intake (38.6 ± 42.1 mg/day) was assessed using a validated self-report questionnaire [13], classifying participants as either caffeine-naive or mild consumers (0−2.99 mg/kg/day) [14]. Inclusion criteria were: (a) healthy men aged 18−35 years; (b) at least two years of resistance training experience, with a minimum of three weekly sessions during the preceding three months; (c) ability to bench press and back squat at least 100% and 125% of body mass, respectively [6]; and (d) no self-reported smoking or caffeine allergy. Each participant signed a written informed consent form. This study adhered to the Declaration of Helsinki and was approved by the Scientific Research Ethics Committee of Shanghai University of Sport (No. 102772023RT203).

### 2.2 Study design

A double-blind, placebo-controlled, randomized crossover design was used. Participants attended the laboratory on three separate occasions. During the first visit, dietary habits, physical activity, and body composition were assessed [15]. An experienced personal trainer then determined each participant's 1RM for bench press and back squat using free weights (Cybex, Medway, MA, USA), following the guidelines of Haff and Triplett [16]. These 1RM values were used to determine individualized loads corresponding to 25%, 50%, 75%, and 90% 1RM for both exercises. Participants were instructed to maintain their regular diet and routines and to abstain from alcohol, caffeine, and strenuous exercise for at least 12 h before each trial.

During visits two and three, participants completed two trials separated by a 7-day wash-out period to allow complete recovery. In one trial, they ingested 4 mg/kg of caffeine (Sigma-Aldrich, Sydney, USA), and in the other, a placebo (4 mg/kg dextrose), both administered as 3−5 identical gelatin capsules per participant [17]. An external researcher assigned alphanumeric codes to the capsules to blind both participants and investigators, with codes revealed only after statistical analysis. Following ingestion, participants rested seated for 60 min to allow caffeine absorption [18]. Trials were performed at the same time of day to minimize circadian effects.

### 2.3 Experimental protocol

One hour after capsule ingestion, participants performed a standardized 10-min warm-up comprising 5 min of self-selected cycling on an ergometer, followed by 5 min of joint mobilization and dynamic stretching. Testing began with 1RM measurements, followed by muscular power measurements at 25%, 50%, 75%, and 90% 1RM during bench press and back squat. Exercise order was counterbalanced across participants and remained consistent within each individual across trials. Two experienced personal trainers supervised to ensure proper technique. Participants were prohibited from wearing weightlifting belts, bench shirts, or other supportive garments during testing.

### 2.4 Maximal strength (1RM) test

Bench press and back squat 1RMs were determined following established protocols [19]. Participants first completed a warm-up of 10 repetitions at 50% 1RM and 5 repetitions at 75% 1RM based on familiarization data [20]. The 1RM was determined within 3−5 attempts, with 5 min of rest between successful attempts [16]. RPE and pain perception were recorded within 5 seconds after each successful lift [21,22]. Pilot testing showed *excellent* test-retest reliability for 1RM measurements, with interclass correlation coefficients (ICC) of 0.98 (95% CI = 0.96−0.99) for bench press and 0.98 (95% CI = 0.95−0.99) for back squat across three days.

### 2.5 Muscular power test

After a 5-min rest following 1RM determination, participants performed bench press and back squat exercises at 25%, 50%, 75%, and 90% of their familiarization 1RM using a 2/0/X/0 cadence (2 seconds for the eccentric phase, no pause during the transition, X representing maximum concentric tempo, and 0 seconds at the end of the movement), paced by a metronome at 30 beats/min [19]. They completed three repetitions at 25% 1RM, two at 50% 1RM, and one each at 75% and 90% 1RM, with 3-min rest intervals. To assess the impact of caffeine on muscular power at different loads, two additional sets were performed post-ingestion at 25% 1RM (3 reps) and 50% 1RM (2 reps). Bar displacement during the concentric phase was recorded using the GymAware Power Testing system (Kinetic Performance Technologies, Canberra, Australia), which has demonstrated *excellent* reliability in measuring velocity and power output [23]. Measured variables included mean velocity (MV in m/s), peak velocity (PV in m/s), mean power output (MPO in W), and peak power output (PPO in W). RPE and pain perception were recorded within 5 seconds after the final repetition. Pilot testing indicated *excellent* test-retest reliability with ICCs for MV (bench press: 0.89 [95%CI = 0.82−0.93]; back squat: 0.89 [95% CI = 0.82−0.94]) and MPO (bench press: 0.96 [95% CI = 0.94−0.98]; back squat: 0.94 [95% CI = 0.91−0.97]) across three days.

### 2.6 Surface electromyography (sEMG)

sEMG data were recorded using a sEMG system (Noraxon, Inc., Scottsdale, AZ, USA) at a 2,000 Hz sampling rate. To ensure consistency across sessions, electrode placements were marked with anatomical pen marks. Skin impedance was minimized by shaving, lightly abrading with sandpaper, and cleaning with alcohol pads. Following SENIAM project's guidelines [24], self-adhesive electrodes (sEMG Electrodes AE−131, NeuroDyne Medical, USA) were placed on the muscle bellies of the dominant side’s *pectoralis major*, *anterior deltoid*, *posterior deltoid*, *biceps brachii*, lateral head of the *triceps* for the bench press, and on the *gluteus maximus*, *rectus femoris*, *vastus medialis*, *vastus lateralis*, *biceps femoris*, and *tibialis anterior* for the back squat. Electrodes were secured using adhesive tape. An electronic goniometer was used to monitor knee and elbow joint flexion and extension, delineating concentric and eccentric phases during each repetition.

Raw sEMG signals were processed using MATLAB (MathWorks Inc., Massachusetts, USA). The concentric phase of each repetition was identified using electronic goniometer data [25] and filtered with a zero-phase, fourth-order Butterworth bandpass filter (10−500 Hz) [26]. A sliding window (0.100 s length, 0.08 s overlap) was employed to calculate RMS values, reflecting motor unit recruitment [27,28]. For the maximal strength test, RMS values were not normalized due to fixed electrode placement and within-subject repeated-measures [29]. For the muscular power test, RMS values at each load were normalized to the peak RMS from the post-ingestion 1RM test [26],with normalized values averaged across repetitions at each load (25%,50%,75%,and 90% 1RM) [26]. Additionally, fast Fourier transform was applied to determine mean frequency (MF) and median frequency (MDF) for both tests [27,30],which reflect muscle recruitment strategy (proportion type I or II muscle fiber recruitment) [31].

### 2.7 Assessment of blinding and side effects

To evaluate blinding effectiveness, participants were asked immediately after capsule ingestion and again post-exercise, *"What substance do you think you ingested?"* with response options: (a) caffeinated capsule; (b) placebo capsule, or (c) I don’t know [32]. Additionally, participants completed a nine-item Side Effects Questionnaire [33], evaluating potential caffeine-related side effects immediately and 24 h after each exercise session.

### 2.8 Statistical analysis

Data were analyzed using SPSS software (Version 22.0; SPSS Inc., Chicago, IL, USA). Normality was confirmed with the Shapiro-Wilk test. Paired sample t-tests were used to compare maximal strength test outcomes (1RM), sEMG variables (RMS, MF, and MDF), and perceptual responses (RPE and pain perception) between caffeine and placebo conditions. A two-way repeated measures ANOVA (load [25%, 50%, 75% and 90% 1RM] × condition [caffeine and placebo]) was performed to analyze the effects of caffeine on exercise outcomes (MV, PV, MPO, and PPO), sEMG variables (RMS, MF, and MDF), and perceptual responses (RPE and pain perception). Partial eta squared (ηp²) values were calculated to estimate effect sizes for main effects, followed by Bonferroni-corrected post hoc tests where applicable. Cohen’s *d* was calculated for pairwise comparisons to assess effect sizes, categorized as *trivial* (<0.20), *small* (0.20−0.49), *moderate* (0.50−0.79), and *large* (≥0.80) [34]. Blinding effectiveness was evaluated with Bang’s Blinding Index (BBI), and McNemar’s test was applied to compare side effects between conditions. Results are presented as mean ± standard deviation (SD), with statistical significance set at *p* < 0.05.

## 3 Results

### 3.1 Maximal strength and sEMG assessment

**Figure 1.**
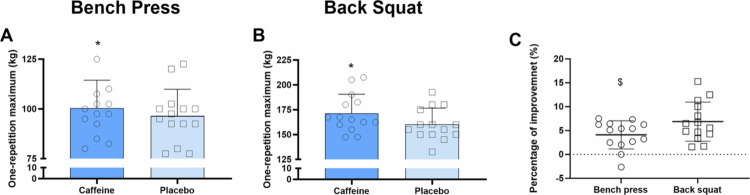
One-repetition maximum in bench press (A) and back squat (B), and percentage improvement (C) in both exercises.Error bars represent standard deviation.* *p < 0.05 vs. placebo, $ p < 0.05 vs. back squat.*

Caffeine significantly increased 1RM for both bench press (+4.1 ± 2.9%, *p* < 0.01, *d =* 0.29) and back squat (+6.9 ± 4.1%, *p* < 0.01, *d =* 0.61) compared to placebo (Figure 1A–B). The improvement was greater for back squat than bench press (+7.0 ± 2.9% vs. + 4.1 ± 2.9%*, p* = 0.03*, d* = 0.53) (Figure 1C). Additionally, caffeine intake enhanced RMS in the *gluteus maximus* (+28.2 ± 30.1%, *p* = 0.05, *d* = 0.61) but had no effect on RMS in other lower limb muscles (all *p* > 0.05) (Table 1). No significant differences were found in MF or MDF between conditions for any muscles (all *p* > 0.05) (Table 1).

**Table 1.** Differences in mean frequency (MF), median frequency (MDF), root mean square (RMS), ratings of perceived exertion (RPE), and pain perception (PP) between caffeine and placebo conditions during maximal strength tests in the bench press and back squat.

Indicators		Caffeine	Placebo
MF (Hz)	**Bench press**		
	pectoralis major	118.99 ± 16.71	116.53 ± 16.06
	anterior deltoid	104.34 ± 14.17	103.60 ± 11.90
	posterior deltoid	104.54 ± 19.99	112.44 ± 16.61
	biceps brachii	104.84 ± 15.36	108.48 ± 10.90
	lateral head of the triceps	118.88 ± 13.83	118.10 ± 16.62
	**Back squat**		
	gluteus maximus	62.56 ± 17.99	63.26 ± 10.35
	rectus femoris	94.38 ± 17.63	93.50 ± 17.06
	vastus medialis	92.20 ± 14.87	96.57 ± 18.45
	vastus lateralis	84.42 ± 12.34	89.31 ± 17.17
	biceps femoris	98.02 ± 21.48	101.48 ± 30.79
	tibialis anterior	145.92 ± 35.51	128.74 ± 26.51
MDF (Hz)	**Bench press**		
	pectoralis major	84.81 ± 19.99	86.98 ± 18.90
	anterior deltoid	76.73 ± 14.47	75.00 ± 12.27
	posterior deltoid	69.82 ± 12.82	77.37 ± 13.48
	biceps brachii	73.16 ± 14.58	74.77 ± 10.62
	lateral head of the triceps	90.55 ± 9.19	88.74 ± 14.69
	**Back squat**		
	gluteus maximus	65.30 ± 12.12	65.49 ± 11.29
	rectus femoris	106.65 ± 15.79	108.09 ± 11.74
	vastus medialis	99.66 ± 16.86	107.16 ± 13.85
	vastus lateralis	86.32 ± 9.84	92.61 ± 14.52
	biceps femoris	99.32 ± 12.85	98.30 ± 23.35
	tibialis anterior	157.47 ± 21.52	153.97 ± 30.00
RMS	**Bench press**		
	pectoralis major	795.26 ± 217.37	711.07 ± 251.08
	anterior deltoid	1594.23 ± 817.28	1569.89 ± 741.76
	posterior deltoid	158.39 ± 64.18	143.12 ± 85.09
	biceps brachii	321.63 ± 322.36	237.13 ± 203.97
	lateral head of the triceps	858.79 ± 435.20	859.04 ± 372.27
	**Back squat**		
	gluteus maximus	527.68 ± 235.47*	417.03 ± 171.86
	rectus femoris	546.43 ± 350.55	496.52 ± 291.80
	vastus medialis	778.38 ± 282.39	725.69 ± 292.95
	vastus lateralis	1052.68 ± 322.97	972.83 ± 371.09
	biceps femoris	351.27 ± 266.62	350.27 ± 200.02
	tibialis anterior	332.47 ± 339.07	238.95 ± 263.56

* *p* < 0.05 vs. placebo.

### 3.2 Muscular power and sEMG assessment

**Figure 2.**
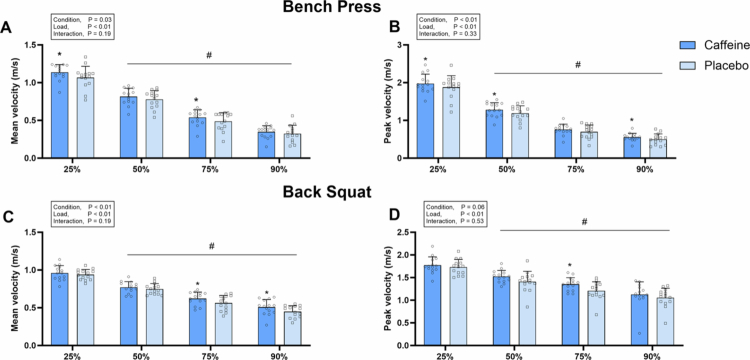
Mean and peak velocity in the bench press (A and B) and back squat (C and D). Error bars represent standard deviation. * p < 0.05 vs. placebo, # p < 0.05 vs. 25% 1RM.

**Figure 3.**
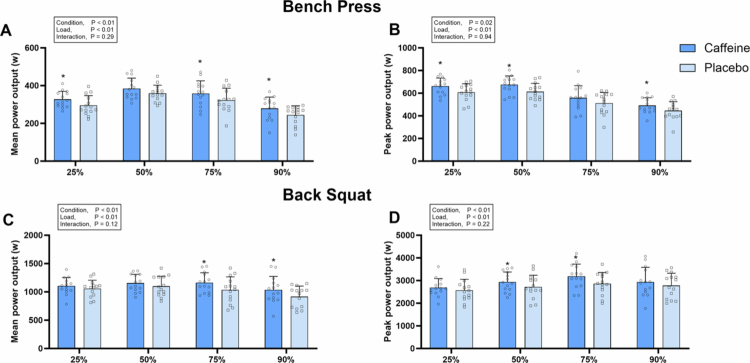
Mean and peak power output in the bench press (A and B) and back squat (C and D) across different percentage of 1RM. Error bars represent standard deviation. * p < 0.05 vs. placebo.

For the bench press, a significant main effect of condition was observed for MV (*F* = 6.213, *p* = 0.03, *ηp²* = 0.32), PV (*F* = 10.498, *p* < 0.01, *ηp²* = 0.65), MPO (*F* = 8.976, *p* = 0.01, *ηp²* = 0.41) and PPO (*F* = 7.368, *p* = 0.02, *ηp²* = 0.36) (Figures 2, 3). Post-hoc comparisons revealed that caffeine increased MV at 25% 1RM (+7.7 ± 8.5%, *p* = 0.01, *d =* 0.60) and 75% 1RM (+13.0 ± 20.1%, *p* = 0.02, *d =* 0.45), and PV at 25%1RM (+6.0 ± 10.7%, *p* = 0.05, *d =* 0.35), 50%1RM (+8.6 ± 11.4%, *p* = 0.02, *d =* 0.49) and 90%1RM (+16.5 ± 26.4%, *p* = 0.04, *d =* 0.44) (Figure 2, 3). It also improved MPO at 25%1RM (+12.0 ± 13.4%, *p* = 0.01, *d =* 0.69), 75%1RM (+8.1 ± 14.2%, *p* = 0.05, *d =* 0.55) and 90%1RM (+14.4 ± 20.6%, *p* < 0.01, *d =* 0.60), and PPO at 25%1RM (+9.9 ± 16.5%, *p* = 0.04, *d =* 0.71), 50%1RM (+11.2 ± 14.1%, *p* = 0.02, *d =* 0.86) and 90%1RM (+13.1 ± 22.3%, *p* = 0.05, *d =* 0.65) (Figures 2, 3). However, no significant differences were found between conditions for RMS, MDF, or MF in any muscles (all *p* > 0.05) (Table 2).

**Table 2.** Differences in mean frequency (MF), median frequency (MDF), root mean square (RMS), ratings of perceived exertion (RPE), and pain perception (PP) between caffeine and placebo conditions during maximal strength tests in the bench press and back squat exercises.

Indicator		25%1RM		50%1RM		75%1RM		90%1RM		*P* value (*pη*^*2*^)		
		Caffeine	Placebo	Caffeine	Placebo	Caffeine	Placebo	Caffeine	Placebo	Condition	Load	Interaction
MF (Hz)	**Bench press**											
	*pectoralis major*	127.7 ± 13.9	130.6 ± 16.2	129.11 ± 13.3	130.7 ± 14.9	128.2 ± 17.7	130.4 ± 14.2	122.7 ± 14.9	129.0 ± 18.6	0.270(0.080)	0.219(0.088)	0.505(0.055)
	*anterior deltoid*	104.9 ± 11.4	103.8 ± 11.4	108.3 ± 12.0	108.4 ± 9.9	105.4 ± 12.9	107.4 ± 8.7	108.2 ± 8.8	107.6 ± 9.1	0.751(0.007)	0.105(0.112)	0.347(0.071)
	*posterior deltoid*	110.2 ± 12.0	110.9 ± 10.4	112.0 ± 11.4	112.4 ± 13.2	113.9 ± 22.0	108.9 ± 14.5	105.6 ± 14.5	110.9 ± 13.9	0.552(0.024)	0.566(0.050)	0.361(0.069)
	*biceps brachii*	106.9 ± 13.1	108.2 ± 11.8	110.9 ± 15.1	110.5 ± 16.1	111.7 ± 13.2	113.7 ± 15.3	107.7 ± 13.9	109.8 ± 21.5	0.865(0.002)	0.168(0.097)	0.953(0.014)
	*lateral head of the triceps*	120.7 ± 12.7	118.3 ± 13.8	120.8 ± 17.2	118.8 ± 15.3	120.2 ± 15.0	119.7 ± 18.8	116.2 ± 15.5	119.6 ± 17.2	0.763(0.006)	0.688(0.039)	0.401(0.065)
	**Back squat**											
	*gluteus maximus*	92.4 ± 13.3	92.8 ± 15.2	94.9 ± 15.4	94.7 ± 20.0	95.5 ± 15.0	100.3 ± 18.0	96.0 ± 13.0	98.0 ± 15.3	0.400(0.048)	<0.01(0.201)	0.661(0.042)
	*rectus femoris*	136.8 ± 16.7	135.1 ± 13.0	135.6 ± 16.4	134.2 ± 15.3	135.5 ± 16.2	131.0 ± 13.0	131.5 ± 14.8	132.5 ± 11.7	0.329(0.063)	0.271(0.080)	0.526(0.053)
	*vastus medialis*	133.1 ± 13.3	128.6 ± 17.5	135.3 ± 15.5	129.8 ± 15.6	130.8 ± 18.3	131.6 ± 21.7	127.8 ± 15.0	133.0 ± 20.0	0.719(0.009)	0.963(0.013)	0.056(0.132)
	*vastus lateralis*	117.0 ± 19.2	116.2 ± 16.8	117.1 ± 15.8	117.4 ± 17.1	116.9 ± 22.0	117.1 ± 19.6	118.1 ± 17.6	113.3 ± 18.2	0.807(0.004)	0.980(0.01)	0.766(0.033)
	*biceps femoris*	123.8 ± 14.3	124.9 ± 18.3	125.3 ± 17.0	129.9 ± 20.4	131.5 ± 18.7	129.6 ± 19.1	130.0 ± 17.3	129.9 ± 22.2	0.757(0.007)	0.076(0.122)	0.758(0.034)
	*tibialis anterior*	171.7 ± 20.7	157.9 ± 20.5	166.1 ± 19.4	161.5 ± 21.6	165.7 ± 21.5	162.1 ± 25.4	159.0 ± 21.2	160.1 ± 19.9	0.157(0.129)	0.453(0.060)	0.239(0.085)
MDF (Hz)	**Bench press**											
	*pectoralis major*	95.2 ± 14.9	97.1 ± 17.5	95.8 ± 14.2	96.4 ± 17.1	93.4 ± 19.4	97.4 ± 16.1	89.4 ± 15.9	94.7 ± 18.3	0.366(0.055)	0.318(0.074)	0.648(0.43)
	*anterior deltoid*	77.9 ± 11.2	76.0 ± 8.8	81.1 ± 11.5	81.2 ± 9.1	79.1 ± 9.8	81.0 ± 8.7	78.2 ± 12.0	80.1 ± 8.3	0.887(0.001)	0.111(0.111)	0.658(0.042)
	*posterior deltoid*	70.7 ± 12.5	72.2 ± 8.1	73.5 ± 13.1	73.7 ± 7.7	76.8 ± 25.4	70.1 ± 14.0	71.4 ± 10.2	71.7 ± 12.6	0.764(0.006)	0.941(0.016)	0.354(0.07)
	*biceps brachii*	70.6 ± 14.8	71.8 ± 13.6	75.9 ± 17.7	76.7 ± 16.3	76.5 ± 15.4	78.2 ± 17.7	75.0 ± 15.8	76.5 ± 19.9	0.903(0.001)	0.034(0.146)	0.953(0.014)
	*lateral head of the triceps*	89.7 ± 13.6	88.7 ± 13.6	90.9 ± 18.3	88.0 ± 14.1	89.9 ± 15.3	88.8 ± 15.3	86.2 ± 14.9	87.7 ± 15.2	0.713(0.009)	0.362(0.069)	0.506(0.055)
	**Back squat**											
	*gluteus maximus*	57.4 ± 14.9	58.3 ± 14.9	59.9 ± 15.4	60.4 ± 17.4	62.3 ± 16.4	64.3 ± 18.3	62.2 ± 10.7	64.9 ± 15.0	0.537 (0.026)	<0.01(0.304)	0.815(0.029)
	*rectus femoris*	99.7 ± 18.5	97.6 ± 14.3	100.4 ± 18.9	98.0 ± 18.8	98.0 ± 21.8	95.7 ± 15.0	98.1 ± 19.1	94.5 ± 10.2	0.259(0.084)	0.179(0.095)	0.800(0.03)
	*vastus medialis*	100.2 ± 14.0	96.9 ± 17.4	105.0 ± 16.5	99.2 ± 16.3	98.1 ± 19.5	98.1 ± 21.5	94.8 ± 14.2	101.6 ± 20.3	0.929(0.001)	0.426(0.062)	0.146(0.102)
	*vastus lateralis*	87.9 ± 18.6	87.3 ± 17.9	91.5 ± 17.1	89.6 ± 16.9	91.8 ± 17.9	91.0 ± 17.7	92.3 ± 16.6	85.8 ± 19.6	0.567(0.022)	0.603(0.046)	0.613(0.046)
	*biceps femoris*	84.4 ± 14.2	83.6 ± 16.8	87.4 ± 16.6	90.9 ± 24.0	94.1 ± 19.9	92.0 ± 17.4	91.9 ± 18.6	93.8 ± 24.1	0.926(0.001)	<0.01(0.212)	0.909(0.02)
	*tibialis anterior*	143.2 ± 25.7	125.1 ± 29.7	135.0 ± 22.0	128.4 ± 31.0	137.1 ± 26.3	130.8 ± 29.9	129.7 ± 27.6	128.7 ± 24.3	0.053(0.227)	0.698(0.039)	0.269(0.08)
RMS	**Bench press**											
	*pectoralis major*	0.651 ± 0.172*	0.512 ± 0.106	0.674 ± 0.161*	0.563 ± 0.137	0.589 ± 0.145	0.652 ± 0.131	0.638 ± 0.126	0.684 ± 0.113	0.052(0.248)	<0.01(0.234)	0.398(0.065)
	*anterior deltoid*	0.683 ± 0.201	0.642 ± 0.161	0.632 ± 0.143	0.712 ± 0.187	0.636 ± 0.097	0.673 ± 0.152	0.74 ± 0.14	0.688 ± 0.157	0.877(0.002)	0.102(0.113)	0.056(0.132)
	*posterior deltoid*	0.557 ± 0.125	0.494 ± 0.173	0.672 ± 0.151	0.647 ± 0.163	0.597 ± 0.132	0.661 ± 0.187	0.655 ± 0.182	0.703 ± 0.166	0.966(0.000)	<0.01(0.454)	0.199(0.091)
	*biceps brachii*	0.651 ± 0.155	0.567 ± 0.132	0.704 ± 0.245	0.697 ± 0.293	0.584 ± 0.141	0.619 ± 0.171	0.774 ± 0.224	0.686 ± 0.121	0.322(0.065)	<0.01(0.251)	0.460(0.059)
	*lateral head of the triceps*	0.552 ± 0.137	0.524 ± 0.186	0.648 ± 0.162	0.612 ± 0.242	0.705 ± 0.184	0.637 ± 0.182	0.752 ± 0.167	0.631 ± 0.142	0.270(0.080)	<0.01(0.454)	0.506(0.055)
	**Back squat**											
	*gluteus maximus*	0.473 ± 0.226	0.492 ± 0.171	0.614 ± 0.224	0.482 ± 0.176	0.623 ± 0.151	0.674 ± 0.215	0.775 ± 0.222	0.721 ± 0.146	0.621(0.017)	<0.01(0.594)	0.160(0.098)
	*rectus femoris*	0.571 ± 0.208	0.507 ± 0.142	0.602 ± 0.209	0.481 ± 0.138	0.671 ± 0.182	0.672 ± 0.166	0.747 ± 0.242	0.592 ± 0.151	0.166(0.124)	<0.01(0.276)	0.140(0.103)
	*vastus medialis*	0.641 ± 0.107	0.693 ± 0.197	0.755 ± 0.162	0.701 ± 0.147	0.823 ± 0.149	0.722 ± 0.152	0.821 ± 0.167	0.712 ± 0.143	0.119(0.154)	<0.01(0.181)	0.045(0.138)
	*vastus lateralis*	0.604 ± 0.162	0.591 ± 0.175	0.693 ± 0.181	0.731 ± 0.227	0.819 ± 0.167	0.726 ± 0.172	0.751 ± 0.187	0.712 ± 0.107	0.624(0.016)	<0.01(0.349)	0.474(0.058)
	*biceps femoris*	0.467 ± 0.208	0.428 ± 0.152	0.581 ± 0.225	0.564 ± 0.232	0.731 ± 0.177	0.653 ± 0.192	0.742 ± 0.171	0.593 ± 0.206	0.201(0.107)	<0.01(0.363)	0.294(0.077)
	*tibialis anterior*	0.438 ± 0.221	0.422 ± 0.212	0.476 ± 0.171	0.561 ± 0.146	0.603 ± 0.225	0.553 ± 0.259	0.57 ± 0.191	0.56 ± 0.236	0.569(0.022)	<0.01(0.214)	0.382(0.067)

MDF: median frequency, MF: mean frequency, RMS: root mean square, RPE: ratings of perceived exertion. Bold text represents statistically significant results.

For the back squat, a significant main effect of condition was observed for MV (F = 11.014, *p* < 0.01, *ηp*² = 0.46), MPO (F = 15.860, *p* < 0.01, *ηp²* = 0.55) and PPO (F = 11.770, *p* < 0.01, *ηp²* = 0.48) (Figures 2, 3). Post-hoc comparisons revealed that caffeine increased MV at 75% 1RM (+12.5 ± 15.4%, *p* < 0.01, *d =* 0.63) and 90% 1RM (+12.3 ± 12.3%, *p* < 0.01, *d =* 0.65). MPO improved at 75% 1RM (+5.4 ± 11.5%, *p* < 0.01, *d =* 0.31) and 90% 1RM (+12.6 ± 13.0%, *p* < 0.01, *d =* 0.57), while PPO increased at 50% 1RM (+8.7 ± 9.6%, *p* = 0.01, *d =* 0.64) and 75% 1RM (+11.8 ± 11.6%, *p* < 0.01, *d =* 0.44) (Figure 2, 3). However, no significant differences were observed in RMS, MDF, or MF for any muscles between conditions (all *p* > 0.05) (Table 2).

**Figure 4.**
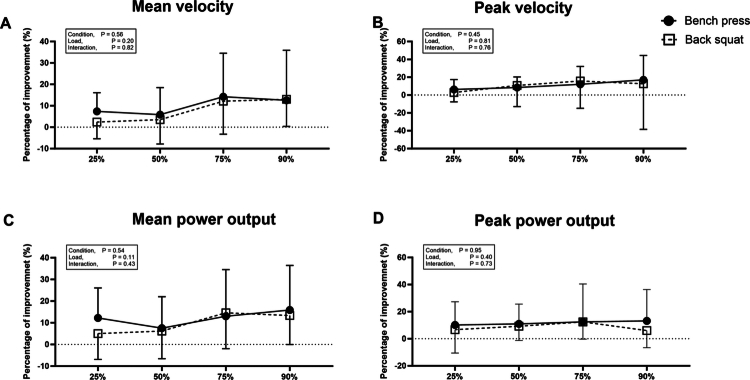
Effect of caffeine ingestion on the percent increase in mean velocity (A), peak velocity (B), mean power output (C) and peak power output (D) during muscular power tests in the bench press and back squat. *Error bars represent standard deviation.*

There was no significant difference between the bench press and back squat in improvements of MV, PV MPO and PPO (all *p* > 0.05) (Figure 4A–D). Additionally, improvements at 50−90% 1RM did not differ significantly from those at 25%1RM for either exercise (all *p* > 0.05) (Figure 4A–D).

### 3.3 Perceptual responses

**Figure 5.**
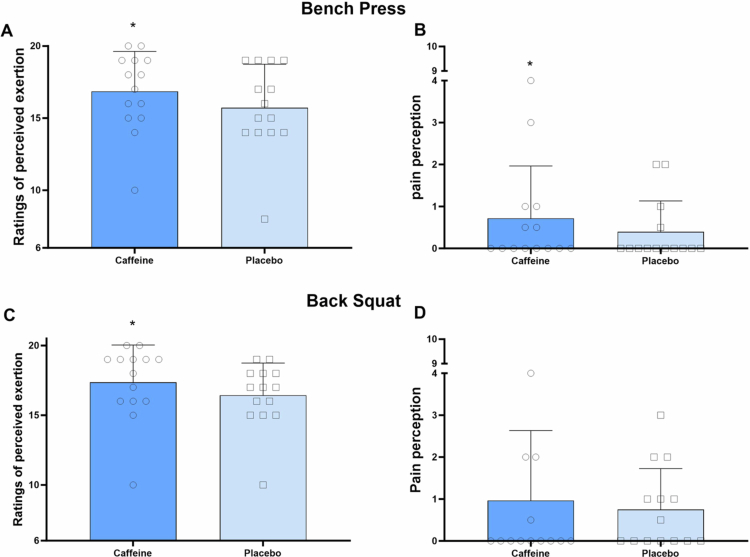
Ratings of perceived exertion and pain perception in the bench press (A and B) and back squat (C and D) during 1RM test. Error bars represent standard deviation. * p < 0.05 vs. placebo.

**Figure 6.**
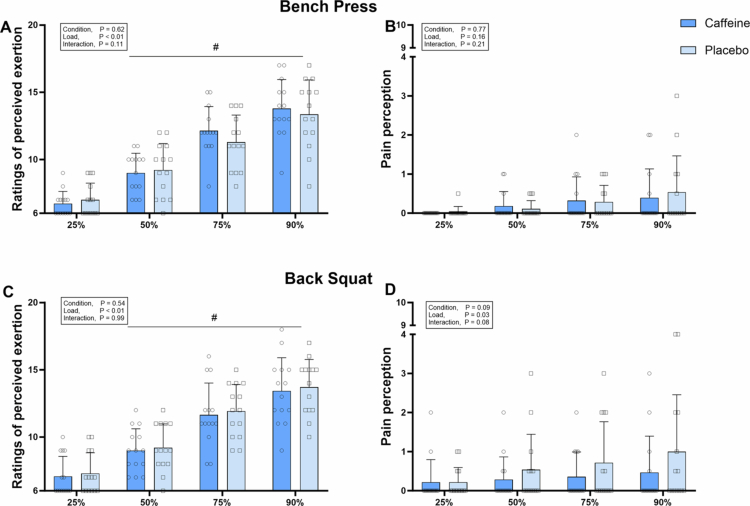
Ratings of perceived exertion and pain perception in the bench press (A and B) and back squat (C and D) during muscular power test. *Error bars represent standard deviation. # p < 0.05 vs. 25% 1RM.*

During the 1RM test, caffeine significantly reduced RPE (16 ± 3 vs. 17 ± 3, *p* < 0.01, *d =* 0.39) and pain perception (0 ± 1 vs. 1 ± 1, *p* = 0.03, *d =* 0.37) for the bench press, and decreased RPE (16 ± 2 vs. 17 ± 3, *p* = 0.02, *d =* 0.37) for the back squat, compared to placebo (Figure 5). However, no significant differences in RPE or pain perception were observed between conditions during the muscular power tests for either exercises (all *p* > 0.05) (Figure 6).

### 3.4 Assessment of blinding and side effects

Blinding was effective immediately following ingestion (caffeine BBI: 0.19 [95%CI: −0.14 − 0.52]; placebo BBI: 0.13 [95%CI: −0.20 − 0.45]) and post-exercise (caffeine BBI: 0.27 [95%CI: −0.13 − 0.66]; placebo BBI: 0.07 [95% CI: −0.11 − 0.24]). Caffeine significantly increased the incidence of *urine output* (57.1% vs. 14.3%, *p* < 0.01), *tachycardia and heart palpitations*(57.1% vs. 14.3%, *p* < 0.01)*, anxiety or nervousness* (28.6% vs. 0%, *p* = 0.03)*, gastrointestinal problems* (42.9% vs. 7.1%, *p* < 0.01)*, increased vigor/activeness* (64.3% vs. 21.4%, *p* < 0.01) and *perception of performance improvement* (42.9% vs. 14.3%, *p* < 0.01) (Table 3). At 24-h post-exercise, caffeine *increased urine output* (28.6% vs. 14.3%, *p* < 0.01), *tachycardia and heart palpitations* (28.6% vs. 0%, *p* = 0.03)*, gastrointestinal problems* (50.0% vs. 14.3%, *p* < 0.01) and *insomnia* (35.7% vs. 7.1%, *p* < 0.01)*,* but did not affect other side effects (e.g. *headache* and *muscle soreness*) (all *p* > 0.05) (Table 3).

**Table 3.** The number (frequency) of participants who reported side effects immediately after and 24 h after testing, out of 16 participants.

Side effect	Caffeine		Placebo	
	+0h	+24h	+0h	+24h
Muscle soreness	5(35.7%)	8(57.1%)	5(35.7%)	7(50.0%)
Increased urine output	8(57.1%)*	4(28.6%)*	2(14.3%)	2(14.3%)
Tachycardia and heart palpitations	8(57.1%)*	4(28.6%)*	2(14.3%)	0(0%)
Anxiety or nervousness	4(28.6%*)	1(6.25%)	0(0%)	0(0%)
Headache	0(0%)	0(0%)	0(0%)	0(0%)
Gastrointestinal problems	6(42.9%)*	7(50.0%)*	1(7.1%)	2(14.3%)
Insomnia	-	5(35.7%)*	-	1(7.1%)
Increased vigor/activeness	9(64.3%)*	3(21.4%)	3(21.4%)	4(28.6%)
Perception of performance improvement	6(42.9%)*	-	2(14.3%)	-

* *p* < 0.05 vs. placebo.

## 4 Discussion

Supporting our hypothesis, caffeine capsule ingestion (4 mg/kg) increased 1RM in both the bench press and back squat by reducing RPE and pain perception, while also improving muscular power without altering these subjective measures. The 1RM improvement was greater for the back squat than the bench press, although no difference in muscular power enhancement was observed between the two exercises. Additionally, caffeine increased RMS activity in the *gluteus maximus* during maximal strength testing, but had no effect on muscle recruitment during muscular power tests. These results suggest that caffeine enhances maximal strength and muscular power via distinct neuromuscular mechanisms.

### 4.1 Effect of caffeine on maximal strength, muscular power, and subjective perception during bench press and back squat

Our results revealed that caffeine improved 1RM during bench press and back squat by alleviating RPE and pain perception (Figure 5). This aligns with previous research showing that caffeine (6 mg/kg) increased back squat 1RM while lowering RPE in resistance-trained individuals [6]. Mechanically, this effect is attributed to caffeine's antagonism of central adenosine receptors, promoting the release of neurotransmitters such as dopamine and ß-endorphin, thereby reducing RPE and pain perception [35,36]. Notably, caffeine also improved muscular power in both exercises without affecting subjective perception (Figure 6). This may be due to the relatively lower loads used in the muscular power tests, which likely induced minimal fatigue and discomfort—factors that are not primary performance limiters in this context. Supporting this, RPE values remained below 15 and pain perception below 1 during both bench press and back squat, regardless of caffeine intake.

#### 4.1.1 Effect of caffeine on maximal strength, muscular power, and muscle recruitment during bench press and back squat

Our findings indicated that caffeine increased 1RM during back squat and elevated RMS activity in the *gluteus maximus* (Table 1), likely attributed to enhanced neuromuscular recruitment via increased central drive [37]. However, caffeine also improved MV, PV, MPO, and PPO without affecting sEMG signals in any of the studied muscles (Table 2). Likewise, San et al [38]. reported that caffeine (3 mg/kg) improved neuromuscular efficiency in elite boxers without affecting EMG activity in the *vastus lateralis*, *gluteus maximus*, or *tibialis anterior* following a 30-second Wingate test. This suggests that, during power-dominant exercises, caffeine likely acts through peripheral mechanisms rather than by enhancing corticospinal drive. For instance, caffeine has been shown to bind to skeletal muscle ryanodine receptor 1, increasing sarcoplasmic reticulum calcium ion release, which promotes cross-bridge formation and enhances maximal strength [39]. However, this proposed mechanism requires further validation through muscle biopsy following resistance exercise.

### 4.2 Difference in maximal strength and muscular power improvements between bench press and back squat

The improvement in 1RM was greater for the back squat than for then bench press (Figure 1). This aligns with previous meta-analysis reporting that caffeine has a more pronounced effect on maximum voluntary contraction strength for larger muscle groups (lower limbs) compared to smaller ones (upper limbs) [40]. One possible explanation is that larger muscle groups such as the knee extensors have greater potential for improvement in voluntary activation levels (typically 85−95%) than smaller muscle groups like the elbow flexors (90−99%) [11]. Our sEMG data further confirmed this hypothesis, showing increased RMS activity in the *gluteus maximus* during back squat, while no significant change were noted in any upper limb muscles during bench press. This indicates that during 1RM tests, caffeine's ergogenic effects are likely driven by central mechanisms (i.e. enhanced more unit recruitment) than peripheral ones. If peripheral mechanisms were dominant, comparable improvements would be expected in both upper and lower limb exercises.

### 4.3 Limitations

This study has three main limitations that should be addressed in future research. First, participants had relatively low habitual caffeine intake (<3 mg/kg/day), which limits the generalizability of our findings to moderate-to-high caffeine consumers (≥3 mg/kg/day), who may exhibit reduced responsiveness due to tolerance [41]. Second, only male participants were included, preventing extrapolation of the findings to female populations. Future research should include more diverse samples to develop more individualized caffeine intake recommendations based on sex, habitual intake, and training background. Finally, the absence of muscle biopsies limited our ability to directly assess peripheral mechanisms (e.g. caffeine induced increases in calcium ion release from sarcoplasmic reticulum).

## 5 Conclusion

Ingesting a caffeine capsule at a dose of 4 mg/kg enhances maximal strength by increasing muscle recruitment and reducing RPE and pain perception, with a larger improvement observed in the bench press compared to the back squat. Additionally, caffeine improves muscular power in both exercises without affecting subjective perception or muscle recruitment.

## Data Availability

The original contributions presented in this study are included in the article. Further inquiries can be directed to the corresponding author.
